# New Magazine of Zoology and Botany

**Published:** 1836-07

**Authors:** 


### NEW MAGAZINE OF ZOOLOGY AND BOTANY.

We call the attention of such of our readers as are interested in the study of natural history (and we trust their number is not small,) to a new journal about to be published on this department of science We agree with the proprietors of the magazine, that such a work is much wanted, and we doubt not that, under such management, it will be both ably and successfully conducted.

" It appears to the proposers of this new periodical that there is at present a great want of some work wherein the progress and discovery of Zoology and Botany can be regularly communicated; that there is now no such work in course of publication, for the many existing periodicals, otherwise excellent, are either devoted to a more extensive range of subjects, or are conducted upon a plan more suited to gain popularity than to advance science; that there is a rising band of young and zealous scientific naturalists, who want some medium for the regular publication of their researches, and whose communications and subscriptions would amply support a magazine conducted upon truly scientific principles. To endeavour to supply this blank in our zoological and botanical literature, the Magazine of Zoology and Botany is proposed. It will be devoted exclusively to those branches of natural science, and will be entirely under the superintendence of Sir W. Jardine, Bart., P. J. Selby, Esq., and Dr. Johnston, of Berwick. It will be published on the first day of every month."

